# Repurposing of NKA inhibitors (‘cardiac glycosides’): a critical analysis

**DOI:** 10.1007/s00210-025-04443-x

**Published:** 2025-07-18

**Authors:** Kayleigh Evans, Roland Seifert

**Affiliations:** Institute of Pharmacology, Hannover Medical School, Carl-Neuberg-Str. 1, D-30625 Hannover, Germany

**Keywords:** NKA inhibitors, Digoxin, Cardiac glycosides, Repurposing

## Abstract

As Na^+^-K^+^-ATPase (NKA) inhibitors (‘cardiac glycosides’) have declining rates of prescriptions because of their narrow therapeutic window, high toxicity and questionable clinical efficacy in cardiological indications, the question arises what the future of these drugs entails. In this paper, we researched and analysed newer uses of these drugs outside of their traditional indications, also referred to as repurposing. On ClinicalTrials.gov, we analysed the trials on NKA inhibitors including the scientific value of published trials. Of 306 trials to be analysed, only 40 were for repurposed use, of which 17 had a publication. Malignant diseases and foetal demise for late abortion are the most prominent indications. However, the quality of the clinical studies analysed was far from satisfying. Not a single study provided compelling evidence for a new clinical indication of NKA inhibitors at clinically relevant drug doses. NKA inhibitors could still have a future, specifically as part of oncological treatments. Different molecular mechanisms of action beyond NKA inhibition may be relevant for this use. However, further research is still required to see if in vitro effects can be translated to in vivo as well, which is the major bottleneck in repurposing NKA inhibitors in their narrow therapeutic window and high toxicity, unless specifically exploited in foetal demise.

**Supplementary Information:**

The online version contains supplementary material available at 10.1007/s00210-025-04443-x.

## Introduction

Repurposing is the development of a known pharmaceutical, which has undergone safety and efficacy testing, for new indications. It is essentially the process of finding new uses for already existing drugs (Begley et al. 2021; Jourdan et al. 2020; Seifert 2021). It is attractive as it decreases drug development costs, increases the rate of drug approval and decreases attrition rate. Furthermore, the adverse effects are already known from the original indication (Begley et al. 2021). The new indication can either be based on a biological property for which a drug has been approved, for example a change in dosage or different administration routes or be linked to the side properties responsible for the adverse effects.

The Na^+^-K^+^-ATPase (NKA) or sodium–potassium pump is an enzyme found in the membrane of every cell and is responsible for the upkeep of the ion gradient. An inhibition of the NKA prevents the exchange of Na^+^ and K^+^, therefore resulting in an increase of intracellular Na^+^ concentrations. This leads to a decrease of the concentration gradient and depletion of energy for the Na^+^/Ca^2+^ exchanger (NCX). This energy depletion causes an accumulation of Ca^2+^ intracellularly. In myocardial cells, this excess Ca^2+^ is stored in the sarcoplasmic reticulum (SR), where it is then released during the systole. This in turn increases contractility and contraction velocity (Blaschek et al. 2007; Karow and Lang-Roth 2023; Lüllmann et al. 2016; WehLing et al. 2011). An increase in vagal tone has also been noted, likely in part due to an increased sensitivity of baroceptors. This increased vagal tone induces the negative dromotropic effect on the atrioventricular (AV) node (Aktories et al. 2017; Lüllmann et al. 2016).

NKA inhibitors can be split into two main groups: the cardenolides and the bufadienolides (Aktories et al. 2017). Both of which are classed as cardiac glycosides, a misleading term as the effects are not solely on myocardial enzymes. For years, they largely dominated the market for heart failure therapy (Seifert 2021). The cardenolides include the digitalis group and strophathin or ouabain group, whereas the main representatives of the bufadienolides are proscillaridin, bufogenin and the endogenous form marinobufagenin (MBG) (David and Shetty 2024; Lüllmann et al. 2016).

The drugs of the digitalis group are digoxin, digitoxin and derivatives of these such as metildigoxin or deslanoside. They all inhibit the NKA leading to the well-known positive inotropic and bathmotropic and negative chronotropic and dromotropic effects, as well as increased diuresis and decreased venous pressure (Ammon and Hunnius 2021; Seifert 2021). The negative dromotropic effect is caused by vagomimetic effects via stimulation of the parasympathetic nervous system which slows the conduction in the AV node. The high intracellular Ca^2+^ levels increase the refractory period of the AV node and therefore decrease ventricular response. This mechanism is why the digitalis group is used in the therapy of atrial fibrillation (AF) (David and Shetty 2024). For all drugs of this group, a therapeutic drug monitoring (TDM) is in place due to a narrow therapeutic window, where toxic concentrations can quickly be reached. Digoxin, for example, has an ideal plasma concentration range of 0.5–0.9 ng/ml or 0.65–1.15 nmol/l (Karow and Lang-Roth 2023).

Cardiac glycosides no longer dominate the market for heart failure therapy, and their prescription rates have declined over the last years. Reasons for this are the narrow therapeutic window and high toxicity, safer and more effective alternatives such as angiotensin-converting enzyme (ACE) inhibitors and β-adrenoreceptor inhibitors, as well as changes in national guidelines (Ludwig et al. 2024; Seifert 2021). As digitalis derivatives are being used less and less for heart failure with reduced ejection fraction (HFrEF) and AF, the question arises if they could possibly be used for alternative indications.

The aim of this study was to discover potential new uses for NKA inhibitors and to summarize the level of research on these drugs. We researched clinical trials relevant to the repurposing of these drugs to see if they still have a future in medicine outside their current indications.

## Materials and methods

**Fig. 1:**
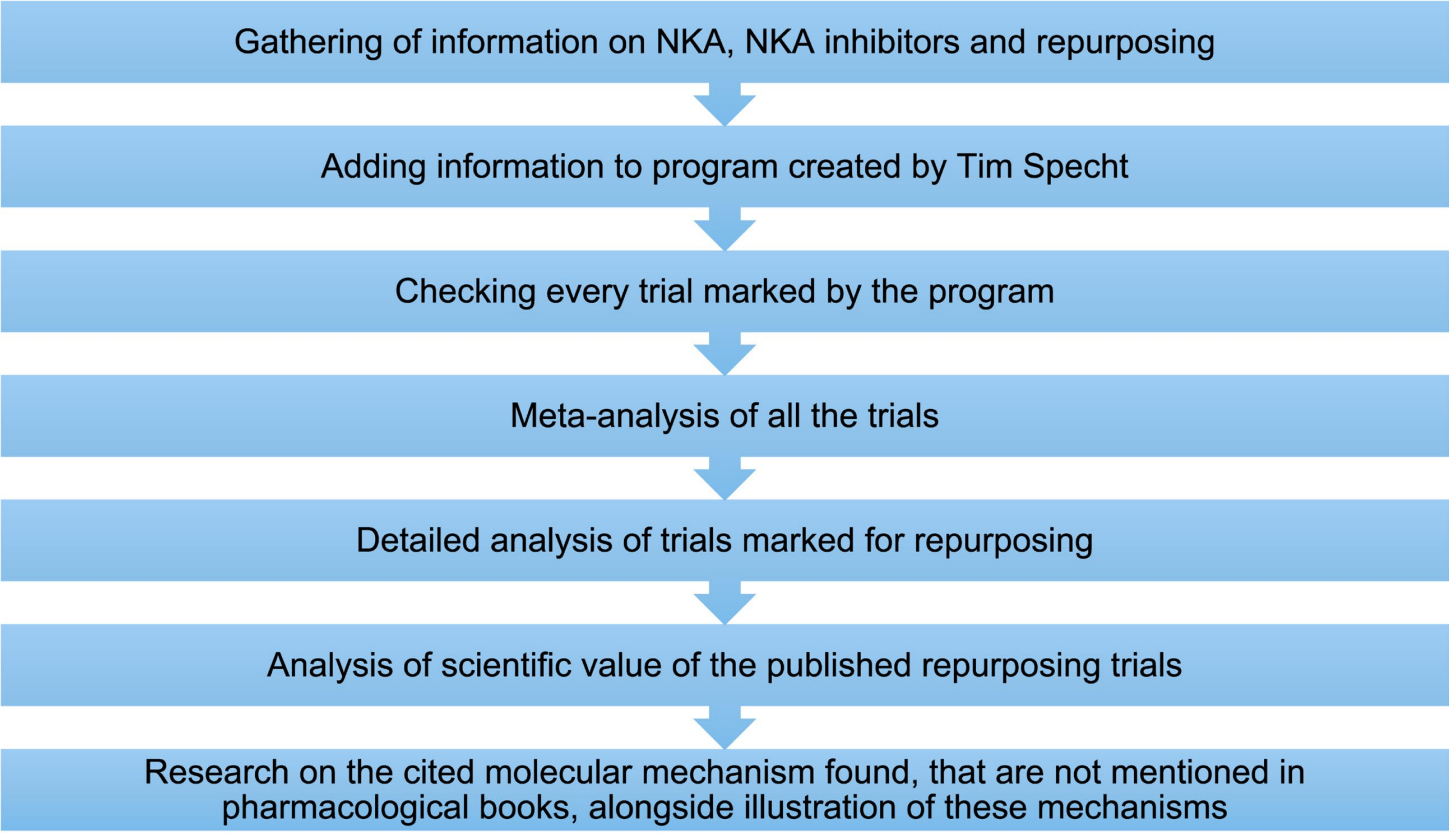
Flowchart of methods employed in analysing identified clinical trials for repurposing. The program in question is defined in ‘Program for analysis’ section Source codes: https://github.com/T-Specht/h1ra-repurpose/ and GitHub - T-Specht/clinical-trials-explorer

The best database for repurposing trials is ClinicalTrials.gov as it is one of the largest databases for clinical trials and studies and lists studies from over 200 countries. Certain trials must be registered by law depending on the country, and in 2006, the World Health Organization (WHO) created a policy that clinical trials all over the world would have to be registered in a database such as ClinicalTrials.gov. The database is set up in a way that sponsors and investigators are responsible for listing information on the trials which can then be accessed by the public (https://clinicaltrials.gov/about-site/about-ctg, last accessed 29 August 2024). After this, any trial we marked as possible repurposing was analysed to the best of our ability with the available information. Figure 1 shows our methods of analysing the trials found.

### Program for analysis

To look through the ClinicalTrials.gov database in search of trials including NKA inhibitors, an open-source program developed by Specht was used. The source code is available on GitHub at https://github.com/T-Specht/h1ra-repurpose/. More information on the program can be found on the webpage or in the paper “Repurposing of H1-receptor antagonists (levo)cetirizine, (des)loratadine, and fexofenadine as a case study for systematic analysis of trials on clinicaltrials.gov using semi-automated processes with custom-coded software” (Specht and Seifert 2023).

In the process of our research, the ClinicalTrials.gov website updated, and Specht created a newer version of the software (https://github.com/T-Specht/clinical-trials-explorer, last accessed 9 December 2024). This newer version was used to check if trials had been updated in the meantime and for data extraction. The main part of the research was conducted using the original software.

**Fig. 2:**
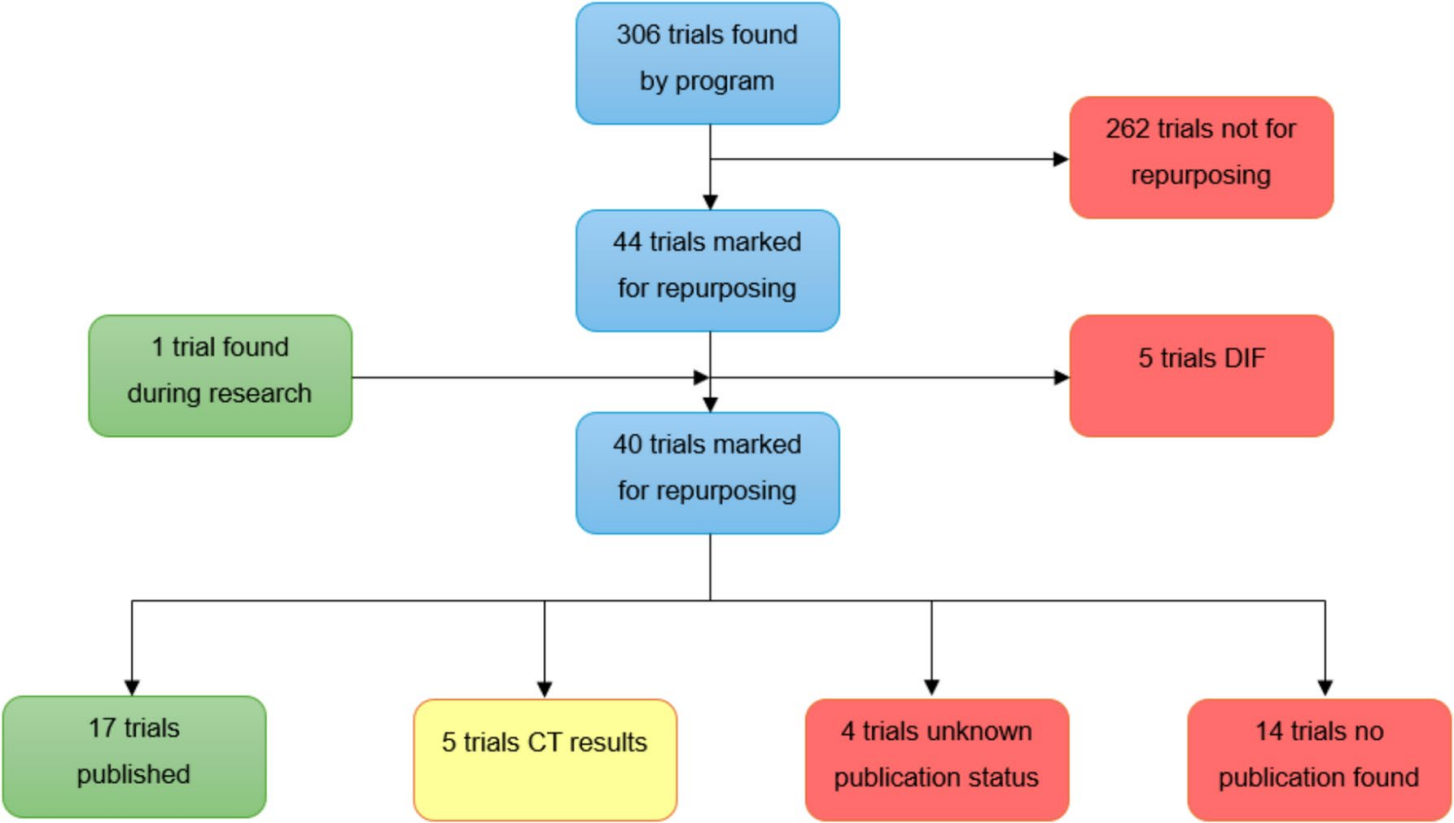
Flowchart of the process of filtering studies regarding repurposing in absolute numbers. Trials marked for repurposing used NKA inhibitors for foreign indications. ‘Unknown publication status’ was used when a trial could not be matched to a publication with certainty. DIF, digoxin immune fab (an antidote for digoxin, not an NKA inhibitor, irrelevant). CT results pertain to results uploaded to ClinicalTrials.gov but not yet published in a paper

After running the program with our search terms deslanoside, digitalis, digoxin, digitoxin, ouabain, strophathin, gitoxin, digitoxinum, metildigoxin and bufadienolides, we obtained 306 trials to analyse. During this process, 262 trials were sorted out as they either did not contain the drugs of interest, study drug interactions, bioavailability and pharmacokinetics or analyse the ‘classic uses’ of the drugs. We were left with 44 trials of which 5 were of digoxin immune fab and therefore not of relevance. Whilst researching a similar trial, we came across another study with a publication containing digoxin as its study drug. This trial was not automatically found and added to the database because ‘digoxin’ is never mentioned or linked in the trial registration but is analysed in its combination under ‘CLS003’. This left us with 40 trials to analyse further of which less than half were published. This process is illustrated in Fig. 2.

## Results

### Data analysis

**Fig. 3:**
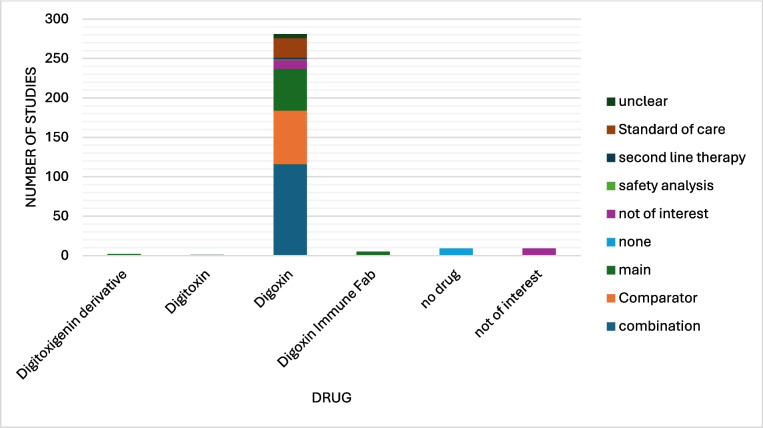
Diagram showing the number of studies related to each drug color coded by the role the drug had in the study in absolute numbers. The drug role was derived from the use of the drug in the study and was not listed as such in ClinicalTrials.gov. Unclear: the role of the drug could not be determined from the study page or possible existing publications. Standard of care: drug was used as a standard of care and was not of interest to the study. Second-line therapy: drug was used as second-line therapy or as a rescue drug and was not a focus of the study. Safety analysis: the safety of the drug was analysed. The drug’s use itself was not focus of the study. Not of interest: NKA inhibitor was mentioned in the abstract, as either an exclusion criterion or as a baseline medication in the patients. They had no role in the study. None: there was no NKA inhibitor in use in the study. Main: NKA inhibitor was the main focus of the study. Comparator: drug was used as an active comparator for a different therapy and was not the main focus of the study. Combination: drug was used in combination with a different drug or therapy

Firstly, we conducted a general analysis of the metadata we had gathered to get an overview of the research state on this topic. Figure 3 shows the number of studies by drug name. For each category, there are between 1 (digitoxin) and 281 (digoxin) results. The category ‘no drug’ (nine studies) was used when the study was strictly observational, and no drugs were distributed to the participants. The category ‘not of interest’ (nine studies) was used mainly for trials which were mistakenly added to the database (five studies) for example because of similar spelling like ‘dioxin’ and ‘digoxin’. In two cases, digoxin was briefly mentioned in the study overview as a medication for heart failure, and in two cases, ouabain was used as a biomarker, this time, however, in an interventional study regarding other medications not relevant to our research.

It is to be mentioned that digoxin immune fab is not an NKA inhibitor, but an antitoxin of digoxin used in acute cases of digoxin toxicity. It is still listed in this study and, in part, has the drug role ‘main’ assigned, as it was part of the analysed studies. Its use, however, would not be analysed in this study.

As one can see, there are many trials that use digoxin, especially as a comparator (68 studies) or in combination (116 studies). This is because digoxin is used for drug interaction testing with other drugs as it is known to be transported by p-glycoprotein. It is therefore commonly used in ‘probe cocktails’ for interaction testing of other drugs (Ebner et al. 2015; Stopfer et al. 2018). It is also to be noted that although digitoxin is most used in Germany as a cardiac glycoside due to the lesser renal strain, especially important in older patients, and economic value, digoxin is more commonly used in other countries such as the USA and the UK. Most research is done with digoxin as the example drug of NKA inhibitors, and not many trials with digitoxin exist (Ludwig et al. 2024). So, it was not surprising that only one trial examining digitoxin was found.

**Fig. 4:**
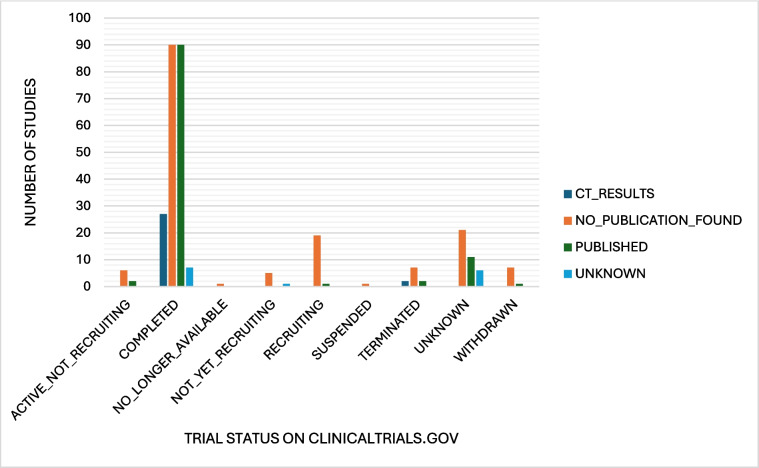
Diagram illustrating the publication status of studies in relation to the status the study was given by ClinicalTrials.gov in absolute numbers. This dataset was last revised on 28 November 2024. It is possible that in the meantime, status and publication status of some of these studies has changed. Active not recruiting: the study is ongoing, and participants are receiving an intervention or being examined, but potential participants are not currently being recruited or enrolled. Completed: the study has ended normally, and participants are no longer being examined or treated (that is, the last participant’s last visit has occurred). No longer available: information on this study is no longer available. Trial has been terminated or withdrawn. Not yet recruiting: the study has not started recruiting participants. Recruiting: the study is currently recruiting participants. Suspended: the study has stopped early but may start again. Terminated: the study has stopped early and will not start again. Participants are no longer being examined or treated. Withdrawn: the study stopped early, before enrolling its first participant. Unknown status is defined above (https://clinicaltrials.gov/study-basics/glossary, last accessed 30 June 2025)

We were then interested to see how the trial status on ClinicalTrials.gov was in comparison to the publication status of these trials (Fig. 4). The trial status ‘unknown’ is “a type of recruitment status. It identifies a study on ClinicalTrials.gov whose last known status was recruiting; not yet recruiting; or active, not yet recruiting, but that has passed its completion date, and the status has not been verified within the past 2 years. Studies with an unknown status are considered closed studies” (https://clinicaltrials.gov/study-basics/glossary, last accessed 13 December 2024). Interesting points of this analysis are the 214 completed trials of which only 90 are published and 27 uploaded results to ClinicalTrials.gov, 90 with no publication found and 7 unknown; then the 8 active, not recruiting trials of which 2 have been published; 11 terminated and 8 withdrawn trials of which together only 3 have been published leaving 16 ‘finished’ trials without publication; and 38 trials with an unknown status. This shows there is inaccurate information regarding the data on ClinicalTrials.gov. The principal investigators are responsible for updating the information of their trials on the website, and when this does not happen, the information is inaccurate and also leads to the ‘unknown’ status. This information gap is particularly problematic for finished trials as the missing information leads to a publication bias within this area of research. Unfortunately, this seems to be common practice for many company-linked trials or trials which were terminated early or withdrawn. The consequence is distortion of the meta-analysis and information readily available for the public and other researchers. On top of this, some trials had uploaded results to ClinicalTrials.gov; however, when thoroughly examined, it was found that no ‘real’ useful information was available as not enough participants were recruited or the ones that were recruited did not take part, etc. So, although results were uploaded, the results contained no useful information and did not deliver any vital information on the success and outcome of the study.

**Fig. 5:**
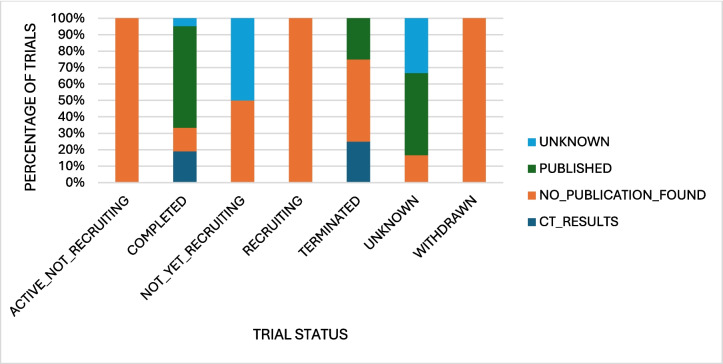
Diagram of the colour-coded publication status of the studies marked ‘repurposing’ in relation to the trial status (*x* axis) stated on the ClinicalTrials.gov website in percentage. The definitions can be found in caption of Fig. 4

With the primary focus of this paper on the repurposing of NKA inhibitors, we performed a more in-depth analysis of the 40 marked trials. Firstly, we analysed the publication status of the trials in comparison to the status listed on ClinicalTrials.gov (Fig. 5) to see if there were any discrepancies. Roughly 62% of the completed trials had a publication and an additional 19% had uploaded results to ClinicalTrials.gov, meaning that information was available for roughly 81% of the trials. Of the terminated and withdrawn trials, there was only information available for roughly 29%, which causes publication bias. Altogether, 85% of the repurposing trials were closed according to ClinicalTrials.gov; however, information was only available for 65% of these. This can be a problem for future research as potentially important information for these trials is not available for other researchers to use and it causes a publication bias in the repurposing research of these drugs.

**Fig. 6:**
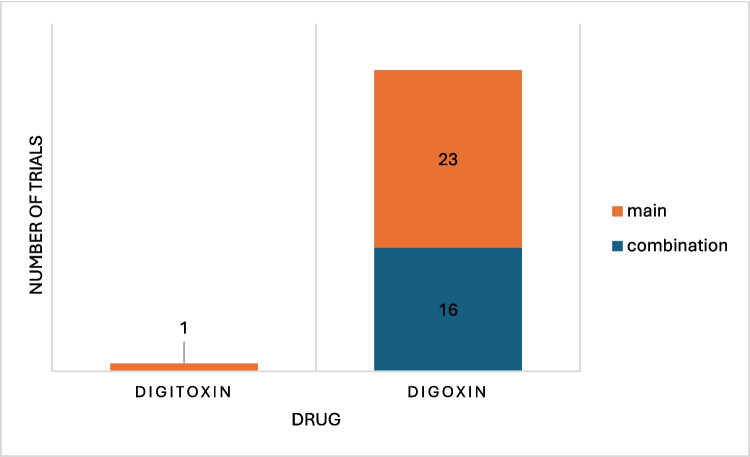
Chart illustrating the role the drug had in the study marked ‘repurposing’ (40 studies) by drug in absolute numbers. The NKA inhibitors examined were digoxin and digitoxin. Main: the drug was the main focus of the study. Combination: the drug was used in combination with other drugs

Furthermore, we were interested to see which NKA inhibitor was used for these trials (Fig. 6). Unsurprisingly, it was digoxin, as this was the main NKA inhibitor being used for other forms of trials as well. However, the single digitoxin trial on ClinicalTrials.gov is a repurposing trial and digitoxin is being used on its own. Interestingly, 41% of the digoxin trials used this drug in combination with at least one other drug. From this, one can deduce digoxin is being used for possible synergistic effects to potentiate efficacy of other drugs or to minimize toxic effects of other drugs. At times, digoxin was used in combination without knowing what the effect of the combination would be, so it was used in an observational way to see if the combination had any positive effects at all and if it was safe.

**Fig. 7:**
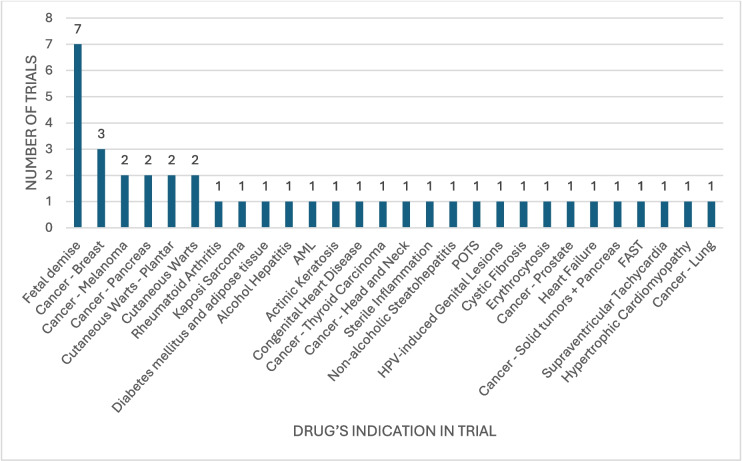
Chart illustrating the different use cases for the ‘repurposed drugs’ in absolute numbers. Use cases were defined during analysis and derived largely from the study’s focus and outcome measures

Following this, we analysed the use cases for the repurposing trials and found 28 different use cases which we sorted into 5 categories (Fig. 7). The largest category was ‘Malignant disease’ followed by ‘Foetal demise for late abortion’, ‘Cardiovascular disease’, ‘Inflammation’ and ‘HPV-induced skin lesions’. A sixth category ‘Other’ exists containing trials that did not fit into any of the other categories and did not create their own summarizing category. The largest category being malignant disease with ten studies of which useable information was available for five studies. Table 1 shows the use cases sorted into categories and whether information was available for these studies. Interestingly, a lot of these use cases are far from NKA inhibitors’ known mechanisms of action and cannot be explained by NKA inhibition.

**Table 1:** Categorical sorting of the use cases of trials involving NKA inhibitors, which were marked as ‘repurposing’

Category	Use case	Reference (NCT number)	Information availability
Malignant disease	1. Cancer (breast × 3) 2. Cancer (melanoma × 2) 3. Cancer (pancreas × 2) 4. Kaposi sarcoma 5. AML 6. Cancer (thyroid carcinoma) 7. Cancer (head and neck) 8. Cancer (prostate) 9. Cancer (solid tumour and pancreas) 10. Cancer (lung)	1. NCT01763931, NCT03928210, NCT01887288 2. NCT02138292, NCT02915666 3. NCT06030622, NCT04141995 4. NCT02212639 5. NCT03113071 6. NCT05507775 7. NCT02906800 8. NCT01162135 9. NCT03889795 10. NCT00281021	1. 1 unknown, 2 with information uploaded to ClinicalTrials.gov (CT results) 2. 1 published, 1 no publication found 3. 2 no publication found 4. Unknown 5. No publication found 6. Published 7. No publication found 8. Published 9. No publication found 10. Published
Foetal demise for late abortion	All 7 foetal demise	NCT03136068, NCT01862991, NCT02277249, NCT01615731, NCT01047748, NCT00382538, NCT01951079	5 published, 2 CT results
Cardiovascular disease	1. Congenital heart disease 2. POTS 3. Heart failure 4. FAST 5. SVT 6. Hypertrophic cardiomyopathy	1. NCT03877965 2. NCT03261570 3. NCT01774656 4. NCT02624765 5. NCT00390546 6. NCT00074880	1. No publication found 2. Published 3. Published 4. No publication found 5. Published 6. No publication found
Inflammation	1. Rheumatoid arthritis 2. Alcohol hepatitis 3. Sterile inflammation 4. Non-alcoholic steatohepatitis 5. Cystic fibrosis	1. NCT04834557 2. NCT05014087 3. NCT03559868 4. NCT04216693 5. NCT00782288	1. Published 2. No publication found 3. CT results 4. No publication found 5. Published
HPV-induced skin lesions	1. Cutaneous warts (plantar × 2) 2. Cutaneous warts × 2 3. HPV-induced genital lesions	1. NCT05599971, NCT05520658 2. NCT02333643, NCT02106260 3. NCT03334240	1. 1 published, 1 unknown 2. 1 published, 1 unknown 3. No publication found
Other	1. Erythrocytosis 2. Actinic keratosis 3. Diabetes mellitus and adipose tissue	1. NCT03433833 2. NCT03684772 3. NCT06240403	1. No publication found 2. Published 3. No publication found

Furthermore, the exact use case, references and information availability of these trials are displayed, alongside the number of trials investigating the use case if there were multiple.

From the 17 published repurposing trials, we analysed different subjects that affect the scientific value of the paper and visualized it in a traffic light system to provide an overview (Table 2). Among the 9 parameters analysed by the traffic light system in the 17 studies, just 59% of the entries fulfilled the criteria of high quality (green), whilst 26% fulfilled the criteria of poor quality (red) and 15% of the entries were neutral (yellow). Not a single study was labelled in green throughout, i.e. no study was ‘perfect’. In contrast, in 4 studies, more than 50% of the parameters were labelled in red, pointing to poor study design/study quality. Thus, the overall quality of the clinical studies analysed is far from satisfying.

**Table 2:** Detailed analysis of published studies on repurposed NKA inhibitors as of 7 December 2024. Factors that decrease scientific value are presented in italics. Factors that increase scientific value are presented in bold. Factors that neither increase nor decrease scientific value are presented in bold–italics. *ITT* intention to treat

Code	NCT	Reference	No. of participants	Randomization	Placebo–control	Masking	Ethical standards	Study type	Sponsor/conflict of interests	Bias/confounding	Journal	Peer review	Evidence level
8	NCT02138292	Frankel et al. (2017)	20	*No*	*No*	*None*	**Followed**	***Prospective, interventional, single cohort, single site***	*Unclear, information not publicly available*	*High likelihood for information bias, selection bias, confounding*	*Neoplasia*^a^	**Yes**	IIb
15	NCT05507775	Van Houten et al. (2024)	7	*No*	*No*	*None*	**Followed**	***Prospective, interventional, single cohort, single site***	***Funded by grant, none declared***	*High likelihood for information bias, confounding*	*European Thyroid Journal*^a^	**Yes**	IIb
29	NCT00782288	Zeitlin et al. (2017)	24	**Yes**	**Yes**	**Triple (participant, care provider, investigator)**	**Followed**	**Prospective interventional, parallel assignment, single site**	*Grants received* *Financial and personal conflicts of interests for corresponding authors*	**Low likelihood for information bias, confounding, selection bias**	*ATS Journals: Annals of the American Thoracic Society*^a^	**Yes**	Ib/IIa (small size)
43	NCT04834557	El-Mahdy et al. (2024)	60	**Yes**	**Yes**	**Double (participant, care provider)**	**Followed; journal is member of Committee on Publication Ethics**	**Prospective, interventional, parallel assignment, single site**	**No funding, no conflicts of interest declared**	**Low likelihood for information bias, confounding, selection bias**	*Frontiers of Pharmacology*^b^	**Yes**	Ib
46	NCT02333643	Rijsbergen et al. (2018)	80	**Yes**	**Yes**	**Double (participant, care provider)**	**Followed**	**Prospective, interventional, parallel assignment, single site, 1:1:1:1 allocation, ITT**	*None declared, 1 co-author associated with sponsor, funded by sponsor*	**Low likelihood for confounding, information bias, selection bias**	*British Journal of Dermatology*^a^	**Yes**	Ib
47	NCT01162135	Lin et al. (2014)	14	*No*	*No*	*None*	**Followed**	***Prospective, interventional, single cohort, single site***	***Funded by grant, none declared***	*High likelihood for confounding, information bias and selection bias*	*American Journal of Cancer Therapy and Pharmacology* (no information on journal found)^b^	*Unknown*	IIb
78	NCT01774656	Birks et al. (2020)	40	*No*	*No*	*None*	**Followed**	***Prospective, interventional, multicentre***	*Grant from pharmaceutical company; many authors with conflicts*	*High likelihood for information bias, selection bias, confounding*	*Circulation from AHA*^a^	**Yes**	IIb
79	NCT01862991	Shaw et al. (2017)	75	**Yes**	**Yes**	**Quadruple (participant, care provider, investigator, outcome assessor)**	**Followed**	**Prospective, interventional, parallel assignment, single site, 1:1:1 allocation**	***Funding by The David and Lucile Packard Foundation, none declared***	***Low likelihood for information bias, selection bias*** ***Middle likelihood for confounding as calculated sample size not reached***	*BJOG: An International Journal of Obstetrics and Gynaecology*^a^	**Yes**	Ib
84	NCT01615731	Shaw et al. (2015)	50	**Yes**	**Yes**	***Single (outcome assessor)***	**Followed**	**Prospective, interventional, parallel assignment, single site**	*Funded by publishing journal, none declared*	*High likelihood for selection bias (randomization failed), information bias* *Middle likelihood for confounding*	*Contraception*^a^	**Yes**	IIa
151	NCT01047748	White et al. (2016)	270	**Yes**	*No*	*None*	**Followed**	**Prospective, interventional, parallel assignment, single centre, 1:1 allocation and ITT**	***None disclosed, funded by grant***	*High likelihood for information bias, confounding* *Low likelihood for selection bias*	*American Journal of Obstetrics and Gynaecology*^a^	**Yes**	IIa
162	NCT00281021	Kayali et al. (2011)	26	*No*	*No*	*None*	**Followed**	***Prospective, interventional, single cohort***	**None disclosed, funding not mentioned**	*High likelihood: selection bias, information bias, confounding*	*Dove Press Open Access Journal for Clinical Trials*^b^	**Yes**	IIb
174	NCT00382538	Kapp et al. (2007)	64	**Yes**	**Yes**	**Double (participant, care provider)**	**Followed**	**Prospective, interventional, parallel assignment, 1:1 allocation**	***Funded by anonymous corporation, none disclosed***	**Low likelihood: selection bias, information bias, confounding**	*American Journal of Obstetrics and Gynaecology*^a^	**Yes**	Ib
179	NCT01951079	Sharvit et al. (2019)	59	*No*	*No*	*None*	**Followed**	***Prospective, single-centre cohort study***	**No disclosure, no specific funding**	*High likelihood: selection bias, information bias, confounding*	*BJOG: An International Journal of Obstetrics & Gynaecology*^a^	**Yes**	IIa/IIb (small size)
229	NCT03261570	Stewart et al. (2021)	56	**Yes**	**Yes**	**Quadruple (participant, care provider, investigator, outcome assessor)**	**Followed**	**Prospective, interventional, crossover with 20 control subjects**	***Funding National Heart, Lung and Blood Institute, none declared***	***Low likelihood: confounding*** ***Middle likelihood: information bias (medication blinding unsuccessful), selection bias (only women)***	*Hypertension from AHA*^a^	**Yes**	Ib
256	NCT03684772	Huismann et al. (2022)	32	**Yes**	**Yes**	**Quadruple (participant, care provider, investigator, outcome assessor)**	**Followed**	**Prospective, interventional, parallel assignment**	*Funding Cutanea Life Sciences, financial and personal conflicts of interest*	***High likelihood for information/publication bias: information supplied by sponsor*** ***Low likelihood: confounding, selection bias, information bias (within study)***	*Journal of the European Academy of Dermatology and Venerology*^a^	**Yes**	IIa
276	NCT00390546	Sanatani et al. (2012)	72	**Yes**	*No*	**Double (participant, care provider)**	**Followed**	**Prospective, interventional, parallel assignment, multicentre**	***No conflicts of interest disclosed, grant funded***	***Low likelihood: selection bias*** ***Middle likelihood: information bias (no full blinding: medication visually different), confounding***	*Circulation: Arrhythmia and Electrophysiology from AHA*^a^	*No*	IIa
307	NCT02106260	Van der Kolk et al. (2017)	12	*No*	*No*	*None*	**Followed**	***Prospective, interventional, single cohort***	***No conflicts of interest stated, funded by Cutanea Life Sciences***	*High likelihood: selection bias, information bias, confounding*	*Journal of the European Academy of Dermatology and Venerology*^a^	**Yes**	IIb

Italics (for red) = factors that decrease scientific value

Journals that increase scientific value are marked with ^a^, journals that decrease this are marked with ^b^

Bold (for green) = factors that increase scientific value

Bold–Italics (for yellow) = factors that neither increase nor decrease scientific value

All studies fulfilled ethical standards; however, only ten studies were randomized of which only eight had a placebo control group. Further, only eight studies were sufficiently masked and one other study had masked only the outcome assessor. Roughly 59% of these studies had a sufficient study design characterised as a prospective, interventional, 2-cohort study with either parallel-assignment or cross-over design. Unfortunately, only three studies were completely free from sponsors or conflicts of interest. Eight further studies had no conflicts of interest to declare, but were sponsored by companies and associations with varying levels of financial and economic gain with positive results. Authors and co-authors of six studies had personal and financial conflicts of interests with their studies. Additionally, we then analysed the likelihood of bias and confounding and split these into three categories: low, middle and high likelihood. We focused on information and selection bias as well as confounding. Confounding can be minimized by randomization, control group and/or matching, stratification and adjusting of results, and age standardization. Therefore, studies not including these had a higher likelihood for confounding in comparison to studies with these characteristics. Lastly, the journals were analysed and journals listed by MEDLINE were marked as increasing the scientific value for the study. MEDLINE follows a rigorous, multi-step process to assess journals and select journals based on five critical elements: scope and coverage, editorial policies and processes, scientific rigor/methodical rigor, production and administration, and impact. The details of this can be found on their website (https://www.nlm.nih.gov/medline/medline_journal_selection.html, last accessed 5 January 2025; National Library of Medicine 2024). For example, for the fiscal year 2024 (1 October–30 September), 37 of 269 journals passed the scientific quality review. This is the reason we used this for assessing the journals. Overall, 14 of the 17 studies were published in journals indexed by MEDLINE. The evidence level of the trials was based on a study by Mehrholz (2010) as seen in Supplementary Table 1. The quality of the trials ranged from Ib to IIb with five trials in category Ib, five in the IIa category and seven in the IIb category. At points where the trial size made it difficult to differentiate between two categories, the lower category was chosen.

## Discussion

During our research into the repurposed uses of NKA inhibitors, we came across different molecular mechanisms, shortly described in Table 3. The diverse mechanisms of action explain why there are so many different studies regarding the use of digoxin ongoing with topics that at first glance do not seem to have any similarities. Additionally, we gathered information on the concentrations of the cardiac glycosides in the different systems to determine whether a translation of experimental results into a clinical setting would even be viable in Table 4. For many experiments, the exact concentration of digoxin or digitoxin in the systems was not listed. A translation of the experimental concentration used into the required plasma concentration was also not listed.

**Table 3:** Pathways affected by NKA inhibitors and their molecular mechanism, as well as possible use

Pathway	Molecular mechanism	Possible uses	References	Findings of clinical trials found
HIF1α	• ROS generation increased with NKA inhibitors; accumulation leads to DNA damage • EPO regulated by HIF, relevant for angiogenesis • Inhibition of HIF and expression of target genes via PKM2 binding and chromatin modifications	• Anticancer therapies • Erythrocytosis	Skubnik et al. (2021) Ouyang et al. (2018) Zhao et al. (2019) Gordeuk et al. (2020) Wang et al. (2020 )	Lin et al. (2014): use of digoxin in prostate cancer: inconclusive results. Digoxin is safe to use at therapeutic dose: inhibition of HIF1α via VEGF measurement inconclusive: inhibition of prostate-specific antigen doubling time comparable to placebo groups. Trial has many limitations
RORγt/NF-κB	• Suppression IκBa phosphorylation blocks access of NF-κB to nucleus: reduced induction of IL-8 • Blocks induction of IL-17 via RORγt and HIF1α: decrease in Th17 differentiation and decrease in IL-17A and IL-23	• Autoimmune diseases • Inflammatory disease • Neuroinflammation	Zeitlin et al. (2017) El-Mahdy et al. (2024) Lee et al. (2015) Saeed et al. (2020)	Zeitlin et al. (2017): use of digitoxin at therapeutic dose in cystic fibrosis (CF) patients is safe, no statistically significant reduction in IL-8 or neutrophile count; however, changes in nasal mRNA indicative of anti-inflammatory effect, suggesting that longer study period could be required for effects on biomarkers El-Mahdy et al. (2024): combination therapy of digoxin and other anti-rheumatic medication for rheumatoid arthritis. Digoxin is safe to use, profound immunomodulatory and anti-inflammatory effects, significantly higher results in digoxin group
NKA (pump): ‘classical’ well-known mechanism	Inhibition of NKA pump: reduced ion transfer, increased intracellular Na^+^ and Ca^2+^, and decreased intracellular K^+^	• Cardiovascular diseases • Cardiotoxicity: foetal demise • Antiviral therapy for DNA viruses • Anticancer therapies	Aktories et al. (2017) Lüllmann et al. (2016) Seifert (2021)	Frankel et al. (2017): BRAF (gene) wild-type metastatic melanoma patients, drug combination, digoxin is safe, achieved high rate of disease control, synergism with MAPK pathway inhibitors. Predominance of stable disease instead of partial remission, however, warrants caution Rijsbergen et al. (2018): HPV-associated skin lesions, digoxin is safe, tested alone and in combination, successful wart reduction and clearance in topical use, best in combination with furosemide Sanatani et al. (2012): trial on SVT prophylaxis in infants comparing digoxin and propranolol, digoxin is safe and efficacious, not significantly better than propranolol, gold standard of treatment
Immunogenic cell death	Induction of ICD with exposure of HSP90, activation of ER stress response, expression of CRT, secretion of ATP and release of nuclear protein high mobility group box 1 (HMGB1)T into extracellular space	• As a senolytic • Anti-inflammatory • Anticancer therapies	Menger et al. (2012)	No published clinical trial investigating this point found NCT06240403 trial ongoing
DNA double-strand break repair	• Inhibition of DNA DSB repair via reduction of expression of DDR proteins • Inhibition of both DSB and SSB	In combination with chemotherapeutics for antiproliferative effect as anticancer therapy	Wang et al. 2020	No clinical trials investigating this mechanism found. Only experimental trials
NIS expression	• Upregulation of NIS expression leads to increase/restoration of iodide uptake • Activation of autophagy and transcription factor FOS (gene)	In combination with radioiodine therapy for thyroid cancer to increase the uptake and therefore potentiate effects	Crezee et al. (2021) Van Houten et al. (2024)	Van Houten et al. (2024): 8 patients with metastasized RAI-refractory NMTC for 3 weeks: digoxin use is safe; despite in vitro success, no reinduction of RAI uptake in all patients at therapeutic dose
NKA (signalling)	• Inhibits TNF-α signalling by interfering with NF-κB translocation • Activation Src/MAPK inhibiting p53 synthesis • Suppression PI3K/Akt pathway: nuclear factor erythroid-derived 2-like 2 (NRF2) inhibition induced activation of Src/EGFR, activates ERK1/2 and increases p21Cip1	Anticancer treatment via multiple different mechanisms such as induced apoptosis, mitochondrial dysfunction and decrease of cell proliferation	Kepp et al. (2012) Wang et al. (2021) Bejček et al. (2021) Liu et al. (2020a) Kayali et al. (2011)	Kayali et al. (2011): combined use for NSCLC with erlotinib, digoxin is safe to use at therapeutic dose, no increase in response rate to treatment Results comparable to erlotinib single use studies Digoxin had no clinical benefit in this study

**Table 4:** Concentration of NKA inhibitor required for effect in the system and in human plasma in comparison to toxic plasma concentrations to determine viability in clinical settings Concentrations converted using a conversion calculator from the website https://unitslab.com/node/176, last accessed 22 April 2025 (Anonymous 2025 ). Plasma concentrations from. Bauersachs et al. (2022)

System	System concentration in vitro/mouse model	Effective plasma concentration in humans	Toxic plasma concentration	Viable in clinical setting?
NKA (pump function)	< 1 nmol/l digoxin (for cardiac effect)	0.64–0.9 nmol/l digoxin (Adams et al. 2016)	Digoxin: > 2.6 nmol/l	Yes
HIF1a	100 nmol/l digoxin (Zhang et al. 2008) < 2.6 nmol/l digoxin (Ouyang et al. 2018)	1.02–2.56 nmol/l digoxin unclear results (Lin et al. 2014)	Digoxin: > 2.6 nmol/l	Data difficult to interpret, more clinical trials required
NKA signalosome	> 2 nmol/l Digoxin (Kometiani et al. 2005) > 5 nmol/l digitoxin (Kometiani et al. 2005)	1.28–2.56 nmol/l digoxin insufficient (Kaya Li et al. 2011)	Digoxin: > 2.6 nmol/l Digitoxin: > 39 nmol/l	Data difficult to interpret, more studies required
Induction of ICD	20–33 nmol/l digitoxin (Guerrero et al. 2019) 100 nmol/l digoxin (Guerrero et al. 2019)	No clinical trial found	Digitoxin: > 39 nmol/l Digoxin: > 2.6 nmol/l	Digitoxin: possible Digoxin: highly unlikely
NIS expression	> 2.6 nmol/l digoxin, exact concentration not given (Crezee et al. 2021)	0.6–1 nmol/l digoxin insufficient (van Houten et al. 2024)	Digoxin: > 2.6 nmol/l	Very unlikely
DNA DSB repair	100–120 nmol/l (Wang et al. 2020)	No clinical trial found	Digoxin: > 2.6 nmol/l	Very unlikely
RORγt/NF-κB	10 nmol/l digoxin (Saeed et al. 2020)	6.5–24.8 nmol/l digitoxin successful (ZeitLin et al. 2017) 1.0–2.56 nmol/l digoxin successful (El-Mahdy et al. 2024)	Digitoxin: > 39 nmol/l Digoxin: > 2.6 nmol/l	Possible

### NKA inhibition

Digoxin has positive inotropic as well as neurohormonal effects including vagomimetic activity, the ability to improve baroreceptor sensitivity, a decrease in norepinephrine serum concentrations and activation of RAAS, a direct sympathoinhibitory effect, and ability to increase release of natriuretic peptides with no effect on the blood pressure (Shiga et al. 2021). These effects allow the drug to be used for versatile cardiovascular diseases such as foetal atrial fibrillation and supraventricular tachycardia (FAST), supraventricular tachycardia (SVT), symptomatic ventricular septum defect (VSD) patients with a left–right-shunt, primary myocardial disease with ventricular dysfunction, for reverse remodelling, as a prophylaxis with cardiotoxic drugs such as anthracycline, and hemodynamic stabilization in interstage period of infants with single ventricular congenital heart disease. The mechanism of action in SVT and FAST is primarily via the enhanced vagal tone inhibiting the atrioventricular node conduction, increasing the refractory period and decreasing conduction velocity, therefore suppressing the heart rate (Birks et al. 2020; Jain and Vaidyanathan 2009; Kumar et al. 2022; Purkayastha et al. 2022; Sanatani et al. 2024; Strasburger et al. 2022). Moreover, its vagotonic actions and improvement of baroreceptor sensitivity have led to the suggestion that it could find use in maintaining the cardiovagal baroreflex. One study by Stewart et al. (2021) tested this hypothesis on postural orthostatic tachycardia syndrome (POTS) patients and found digoxin did indeed repair the supine cardiovagal baroreflex; however, it was not able to maintain it in an upright position. It is now being tested in combination with physical exercise/reconditioning (Stewart et al. 2021).

Digitalis toxicity is generally an effect physicians want to avoid. However, in one instance, it is desired. In high concentrations, it is cardiotoxic due to the electrolyte imbalances it induces, mainly hyperkalaemia and hypercalcemia. This causes arrythmia, AV blockages, sinus bradycardia and sinus arrest. In its use as a fetotoxic agent for second-trimester abortions, 1 mg of digoxin is injected intra-amniotically or intrafoetally. This is a common practice in the USA. In Germany and other countries, potassium chloride (KCl) is used as a fetotoxic agent (Diedrich et al. 2020; Drey et al. 2000; Gariepy et al. 2013; Jackson et al. 2001; Shaw et al. 2015; Tufa et al. 2020a, 2020b). The WHO recommends foetal demise to be considered when terminating a pregnancy beyond 20 weeks, whereas the Royal College of Obstetricians and Gynaecologists recommend the induction for medical abortions > 21 + 6 weeks. This is usually done 24 h prior to the dilation and evacuation procedure (Diedrich et al. 2020; Molaei et al. 2008; Sharvit et al. 2019; Tocce et al. 2013; White et al. 2016). So, in this instance, the toxic effects of a drug overdose are desired.

Focusing on the same molecular mechanism digoxin may also be used for antiviral therapies. The reasoning behind this is that the inhibition of the NKA decreases the K^+^ influx into the intracellular space. K^+^ is vital for the activity of the deoxyribonucleic acid (DNA) polymerase and therefore for DNA replication, for example for the human papilloma virus (HPV) (Bernal Masferrer et al. 2024; Hartley et al. 2006; Huisman et al. 2022; Khattab et al. 2024; Lofty et al. 2022; Rijsbergen et al. 2018; van der Kolk et al. 2017). Trials by Rijsbergen et al. (2018) and van der Kolk et al. (2017) tested this theory with a randomized controlled trial (RCT) testing digoxin and furosemide alone and in combination and found that digoxin alone reduced wart size and HP viral load in its use as a topical gel. In combination with furosemide, the positive effects were significantly greater. These results are promising.

Inhibition of NKA disturbs the intracellular ion distribution and the potential of the cellular membrane, altering cellular homeostasis and inducing apoptosis and increased sensitivity to toxins and cellular stress (KayaLi et al. 2011). Additionally, the NKA inhibition–mediated Ca^2+^ level elevation can suppress cell–cell junctions (required to disaggregate CTC clusters) and cause mitochondrial-mediated tumour apoptosis. This inhibits the epithelial-to-mesenchymal transition (EMT) process inhibiting migration and invasion of tumour cells (Li et al. 2021; Schuster et al. 2021). Both these mechanisms together form a basis for the drug’s use in malignant diseases.

### Signalosome

The NKA functions not only as a pump but also as a signalosome coupled with an enzyme of the Src family of nonreceptor tyrosine kinases. Together, they build a functional complex for signal transduction (Bejček et al. 2021). One pathway that is activated is the complex of NKA, SrcK and epidermal growth factor receptor (EGFR), which leads to the dissociation of SrcK which in turn phosphorylates EGFR and the subsequent cascade stimulates cell cycle progression and cell proliferation (Bejček et al. 2021; KayaLi et al. 2011; Škubník et al. 2021; Wicks and Semenza 2022). Depending on cell type and NKA isoform, healthy cell proliferation is stimulated and tumour cell proliferation is inhibited, leading to a selective reduction of cancer cell proliferation. Human cancer cells are assumed to express particular isoforms of the NKA subunits making them more sensitive to NKA inhibitors (Bejček et al. 2021; Menger et al. 2012).

Another involved pathway is with nuclear factor kappa-light-chain-enhancer of activated B cells (NF-κB). The NKA/SrcK signalosome activates the phospholipase C which produces inositol triphosphate (IP3) to interact with its receptor. This then stimulates Ca^2+^ oscillation and induces the synthesis of the antiapoptotic subunit p65 of NF-κB. NF-κB is a specific transcription factor that regulates cell proliferation, immune response and apoptosis. It activates the calmodulin-dependent kinase 2G, which inactivates the proapoptotic protein BAD (BCL-2-associated agonist of cell death protein) (Bejček et al. 2021; Wang et al. 2020). Moreover, digoxin can interfere with NF-κB translocation interfering with the tumour necrosis factor alpha (TNF-α) signalling (Kepp et al. 2012). Cardiac glycosides reduce p53 levels by initiating Src/mitogen-activated protein kinase (MAPK) signalling pathways and inhibiting the p53 synthesis (Liu et al. 2020a, b). These signalling pathway interferences could be relevant for the effects of digoxin in malignant diseases. Kayali et al. (2011) tested the use of digoxin in combination with a chemotherapeutic agent for non-small cell lung carcinoma (NSCLC) and found the use to be safe, but it had no significant effect on the disease and outcome.

### Hypoxia-inducible factor

Hypoxia-inducible factor (HIF) is a heterodimeric protein with various isoforms of subunits, the most common being HIF1α. HIF plays a crucial role in the regulation of cellular oxygen homeostasis, including angiogenesis, glycolysis and cell proliferation (Supplementary Fig. 1) (Gordeuk et al. 2020; Qannita et al. 2024; Wicks and Semenza 2022; Zhang et al. 2008). In hypoxic conditions HIF binds hypoxic response elements (HREs) which activate the transcription of genes involved in cell growth, cell survival and angiogenesis (Yehia et al. 2015). HRE activation results in the transcription of erythropoietin (EPO), vascular endothelial growth factor (VEGF) and RAR-related orphan receptor gamma (RORγt) among other genes (El-Mahdy et al. 2024; Gordeuk et al. 2020; Škubník et al. 2021). In the case of cancer, the consequence of HIF activation in the hypoxic tumour is an increased tumour vascularization, cell motility and invasion via facilitation of EMT, resistance to chemo- and radiation therapy, cancer stem cell specification and immune evasion (Qannita et al. 2024; Wicks and Semenza 2022; Zhang et al. 2008).

Digoxin inhibits protein biosynthesis of HIF1α and expression of its target genes in cancer cells (Wang et al. 2020; Wicks and Semenza 2022; Zhang et al. 2008). It is assumed to accomplish this by binding pyruvate-kinase muscle isozyme 2 (PKM2). As PKM2 interacts with HIF1α in the nucleus and functions as a transcriptional coactivator, there is a reduced upregulation of HIF1α (Jamshed et al. 2023; Ouyang et al. 2018; Škubník et al. 2021; Zhao et al. 2019). Subsequently, there is a decrease in transcription of target genes such as VEGF reducing invasive capabilities of tumour cells and slower angiogenesis (Bejček et al. 2021; Ring et al. 2022; Schuster et al. 2021).

Lin et al. (2014) tested digoxin in the patients with advanced stage prostate cancer. To analyse the effect on HIF1α the concentration of VEGF was measured. VEGF, however, was only detectable in four patients at baseline. During the trial VEGF rose continuously in 2 patients and declined to not be measurable in the other 2. Due to the very small patient sample the results are inconclusive and insufficient in measuring digoxin’s ability to inhibit HIF1α. There has been no rerun of the trial.

### Immune cells and apoptosis

NKA inhibitors have been linked to influencing immune cells and inflammation, specifically the Th17 differentiation (Supplementary Fig. 2). Digoxin and digitoxin effectively reduce Th17 differentiation and associated inflammation by inhibiting key pathways (NF-κB, STAT3 (Signal transducer and activator of transcription 3), HIF1α, and RORγt) (Lee et al. 2015; ZeitLin et al. 2017). Digoxin has been found to decrease the messenger ribonucleic acid (mRNA) expression of RORγt and also inhibit HIF1α, which activates RORγt transcription. Consequently, interleukin (IL)−17 decreases and therefore also Th17 differentiation. These effects render NKA inhibitors candidate drugs for managing autoimmune, inflammatory, and metabolic disorders.

Zeitlin et al. (2017) tested IL-8 and neutrophil count as inflammatory biomarkers in the sputum of patients with cystic fibrosis. Then the group was split into control and digitoxin group and after the treatment phase the values were measured again. They found no statistically significant decrease in IL-8 or neutrophil count in the digitoxin group. However, from the nasal swabs changes in mRNA indicative of anti-inflammatory response were noted. El-Mahdy et al. (2024) ran a trial adding digoxin to a drug combination for rheumatoid arthritis and analysed its effects. In the digoxin group there were significantly higher anti-inflammatory and immunomodulatory effects in comparison to the control group.

NKA inhibitors induce apoptosis via immunogenic cell death (ICD), inhibition of double-strand break (DSB) and modulation of autophagy (Crezee et al. 2021). DNA DSB repair is part of the cellular DNA damage repair (DDR) network crucial to maintain survival and proliferation of cancer cells and is commonly targeted in oncological treatments. Digoxin inhibits DNA DSB and single-strand break (SSB) repair and potentiates the induction of DNA damage by ionizing radiation and cytostatic drugs (Wang et al. 2020). Via ICD, digoxin mechanism they can ameliorate efficacy of non-immunogenic anticancer therapies (Bejček et al. 2021; Menger et al. 2012; Škubník et al. 2021). These effects have yet to be tested in clinical trials.

### Digoxin in clinical trials for malignant diseases

(Frankel et al. 2017) investigated the use of digoxin in combination with MEK (MAPK/extracellular signal-regulated kinase (ERK)) inhibitor trametinib for advanced melanoma. The effects in the combination group were significantly greater than trametinib alone, however, partial regression was only achieved in 20% of patients and 65% of patients exhibited stable disease. Whereas this study does show some positive results, this was a small study cohort and disease control is not the same as disease regression. This phase 1b trial is successful enough to warrant an advancement onto a phase 2 trial of digoxin and MEK inhibitors in combination for malignant disease.

Lin et al. (2014) tested digoxin in patients with biochemically relapsed prostate cancer (PCa). Patients received digoxin till the target therapeutic level of 0.8–2 ng/ml was reached and continued for at least 6 months. Although digoxin was deemed safe to use at this concentration, there was no significant difference in disease progression in comparison to historical placebo control data.

Kayali et al. (2011) investigated the use of digoxin in combination with erlotinib in NSCLC as in vitro results of digoxin had been promising. During the trial, 24 patients completed the 6-week treatment period with digoxin levels kept at therapeutic levels. The trial was terminated at interim analysis as the results were comparable to erlotinib alone.

Van Houten et al. (2024) trialled digoxin for its use in non-medullary thyroid carcinoma (NMTC). Preclinical trials had been promising of digoxin restoring the radioactive iodine (RAI) uptake which in turn would have improved prognosis. Seven patients participated in the 3-week trial, and none of the patients showed clinically relevant RAI uptake levels after treatment. Although the trial results could be seen as negative, this trial helped steer investigation away from digoxin.

### Dosage and plasma concentration in comparison

From Table 4, it can be deduced that digoxin’s effect on sodium iodide symporter (NIS) expression, DNA DSB repair and induction of ICD will very unlikely find practical use in a clinical setting due to the extremely high plasma concentrations required. Table 4 also shows the toxic plasma concentrations of NKA inhibitors (Bauersachs et al. 2022). If we adhere to these definitions, then only the inhibition of the pumping function of the NKA would be reached. Accordingly, we then analysed the dosage used in these clinical trials and compared them to the dosage in cardiac conditions. Nearly all the dosing was within the limits of the current dosing (Table 5). This is because all principal investigators aimed to maintain digoxin levels within their therapeutic range and not induce digitalis toxicity as this could be lethal. The only times this was not followed were when digoxin was used as a feticidal agent and the toxic effect was desired. There is little data available on the uptake of digoxin over the skin, so that the dosing of gels likely proves to be more difficult; however, in the dosage used, there were no adverse effects associated with digoxin.

**Table 5:** Dosing of digoxin in clinical trials in comparison to dosing for cardiac indications Loading dose and maintenance dose based on Bauersachs et al. (2022)

Clinical trial	Trial result based on primary outcome	Dosage used	Loading dose over 3 days for cardiac indications	Maintenance dose for cardiac indications	Dosage in line with current dosing?
**NCT02138292 (**Frankel et al. 2017**)**	Positive trial result	0.25 mg digoxin without loading	Digoxin p.o./i.v.: 0.75–1.5 mg	Digoxin p.o.: 0.05–0.25 mg	Yes
**NCT05507775 (**van Houten et al. 2024**)**	Negative trial result	0.125–0.375 mg with TDM and without loading dose	Digoxin p.o./i.v.: 0.75–1.5 mg	Digoxin p.o.: 0.05–0.25 mg	Yes
**NCT00782288 (**ZeitLin et al. 2017**)**	Inconclusive trial result	0.05–0.1 mg digitoxin without loading dose	Digitoxin p.o./i.v.: 0.5–1.0 mg	Digitoxin p.o.: 0.035–0.1 mg	Yes
**NCT04834557 (** El-Mahdy et al. 2024**)**	Positive trial result	0.25 mg digoxin every other day, no loading	Digoxin p.o./i.v.: 0.75–1.5 mg	Digoxin p.o.: 0.05–0.25 mg	Yes
**NCT02333643 (** Rijsbergen et al. 2018**)**	Positive trial result	0.125% w/w digoxin in topical gel	Digoxin p.o./i.v.: 0.75–1.5 mg	Digoxin p.o.: 0.05–0.25 mg	Dosage dependent on amount of gel applied
**NCT01162135 (**Lin et al. 2014**)**	Negative trial result	0.25 mg first week, then 0.125 mg following weeks	Digoxin p.o./i.v.: 0.75–1.5 mg	Digoxin p.o.: 0.05–0.25 mg	Yes
**NCT01774656 (**Birks et al. 2020**)**	Positive trial result	0.125 mg digoxin, without loading	Digoxin p.o./i.v.: 0.75–1.5 mg	Digoxin p.o.: 0.05–0.25 mg	Yes
**NCT01862991 (Shaw et al. **2017**)**	Positive trial result	1 mg digoxin intra-amniotically	Digoxin p.o./i.v.: 0.75–1.5 mg	Digoxin p.o.: 0.05–0.25 mg	No, but effect is desired
**NCT01615731 (Shaw et al. **2015**)**	Positive trial result	1 mg digoxin intra-amniotically	Digoxin p.o./i.v.: 0.75–1.5 mg	Digoxin p.o.: 0.05–0.25 mg	No, but effect is desired
**NCT01047748 (**White et al. 2016**)**	Positive trial result	1 mg digoxin intrafoetally and intra-amniotically	Digoxin p.o./i.v.: 0.75–1.5 mg	Digoxin p.o.: 0.05–0.25 mg	No, but effect is desired
**NCT00281021 (**Kaya Li et al. 2011**)**	Negative trial result	0.25 mg digoxin with TDM	Digoxin p.o./i.v.: 0.75–1.5 mg	Digoxin p.o.: 0.05–0.25 mg	Yes
**NCT00382538 (Kapp et al. **2007**)**	Positive trial result	1.5 mg digoxin intra-amniotically	Digoxin p.o./i.v.: 0.75–1.5 mg	Digoxin p.o.: 0.05–0.25 mg	No, but effect is desired
**NCT01951079 (**Sharvit et al. 2019**)**	Positive trial result	2 mg digoxin intra-amniotically	Digoxin p.o./i.v.: 0.75–1.5 mg	Digoxin p.o.: 0.05–0.25 mg	No, but effect is desired
**NCT03261570 (**Stewart et al. 2021**)**	Positive trial result	0.5 mg digoxin every other day	Digoxin p.o./i.v.: 0.75–1.5 mg	Digoxin p.o.: 0.05–0.25 mg	Yes
**NCT03684772 **(Huismann et al. 2022**)**	Negative trial result	0.125% w/w digoxin in topical gel	Digoxin p.o./i.v.: 0.75–1.5 mg	Digoxin p.o.: 0.05–0.25 mg	Dosage dependent on amount of gel applied
**NCT00390546 (Sanatani et al.** 2012**)**	Inconclusive trial result	0.010 mg/kg per dose TID, then 0.0035 mg/kg per dose TID for the third and subsequent doses	Digoxin dose weight dependent with higher loading dose, reduced to maintenance over 3 days	Digoxin dose weight dependent: for 3 kg newborn: 0.3 mg p.o. and 0.2 mg i.v. (Ankermann et al. 2020)	Dosage dependent on age and weight of child
**NCT02106260 (**van der Kolk et al. 2017**)**	Positive trial result	0.125% w/w digoxin in topical gel	Digoxin p.o./i.v.: 0.75–1.5 mg	Digoxin p.o.: 0.05–0.25 mg	Dosage dependent on amount of gel applied

## Conclusions

There are currently many ongoing studies examining the uses of NKA inhibitors (mainly digoxin) for repurposed indications, and new data on the different molecular mechanisms open more possibilities of use of NKA inhibitors. However, to this end, convincing evidence for a new clinical use of NKA inhibitors within the narrow therapeutic window is missing. The scientific value of the clinical studies conducted so far is limited, and unfortunately, there is a lot of information missing. NKA inhibitors have already established their use as feticidal agents in the USA for second-trimester abortion, but here, the toxicity is actually the desired effect.

## Limitations and future studies

There are limitations to our study, the most prominent being the continuously changing database of trials and updates on ClinicalTrials.gov. However, we have tried to minimize the time since last update and analysis as much as possible. Additionally, there is the publication bias which we already mentioned during our analysis as there are results of trials missing and therefore not available for us to analyse. The repurposing of NKA inhibitors is an area worth continuous research, but the bottleneck will be to remain within the narrow therapeutic window without inducing toxicity. This bottleneck actually curtailed the clinical use of NKA inhibitors in cardiological indications.

## Supplementary Information

Below is the link to the electronic supplementary material.

Supplementary file (DOCX 164 KB)

## Data Availability

All source data for this study are available upon reasonable request from the authors.

## Abbreviations

AML: Acute myeloid leukaemia
ATP: Adenosine triphosphate
BCL: B-cell lymphoma
Ca^2+^: Calcium
CRT: Calreticulin
CTC: Circulating tumour cell
DSB: Double-strand break
EGFR: Epidermal growth factor receptor
EMT: Epithelial-to-mesenchymal transition
EPO: Erythropoietin
ER: Endoplasmic reticulum
FAST: Foetal atrial fibrillation and supraventricular tachycardia
HIF: Hypoxia-inducible factor
HPV: Human papilloma virus
HRE: Heat response element
HSP90: Heat shock protein 90
ICD: Immunogenic cell death
IL: Interleukin
K^+^: Potassium
MAPK: Mitogen-activated protein kinase
mRNA: Messenger ribonucleic acid
Na^+^: Sodium
NF-κB: Nuclear factor kappa-light-chain-enhancer of activated B cells
NIS: Sodium iodide symporter
NKA: Na^+^-K^+^-ATPase
NMTC: Non-medullary thyroid carcinoma
NSCLC: Non-small cell lung carcinoma
PKM2: Pyruvate-kinase muscle isozyme 2
POTS: Postural orthostatic tachycardia syndrome
RAAS: Renin–angiotensin–aldosterone system
RAI: Radioactive iodine
RORγt: RAR-related orphan receptor gamma
ROS: Reactive oxygen species
SSB: Single-strand break
TDM: Therapeutic drug monitoring
VEGF: Vascular endothelial growth factor
