# Supplementary material for: Repurposing of NKA inhibitors (‘cardiac glycosides’): a critical analysis
Source: Naunyn Schmiedebergs Arch Pharmacol. 2025 Jul 18;399(1):721–44. doi: 10.1007/s00210-025-04443-x (PMC12894119; doi:10.1007/s00210-025-04443-x)
Supplement: Supplementary file 1 — Supplementary file (DOCX 164 KB) [file 210_2025_4443_MOESM1_ESM.docx]

**Repurposing of NKA inhibitors (“cardiac glycosides”: A critical analysis**

**Kayleigh Evans and Roland Seifert**

Supplemental tables:

Table S1: Evidence level criteria used for published studies based on Mehrholz, 2010.

| Evidence level | Description |
| --- | --- |
| Ia | Systematic review based on high-quality randomized controlled trials (RCT) |
| Ib | A high-quality RCT with sufficiently large size |
| IIa | A high-quality study without randomization, i.e. a cohort study |
| IIb | A high-quality study of a different type of quasi-experimental / non-randomized study |
| III | A high-quality non-experimental study |
| IV | Expert’s opinion, Expert commission or descriptive study |

Supplemental figures:


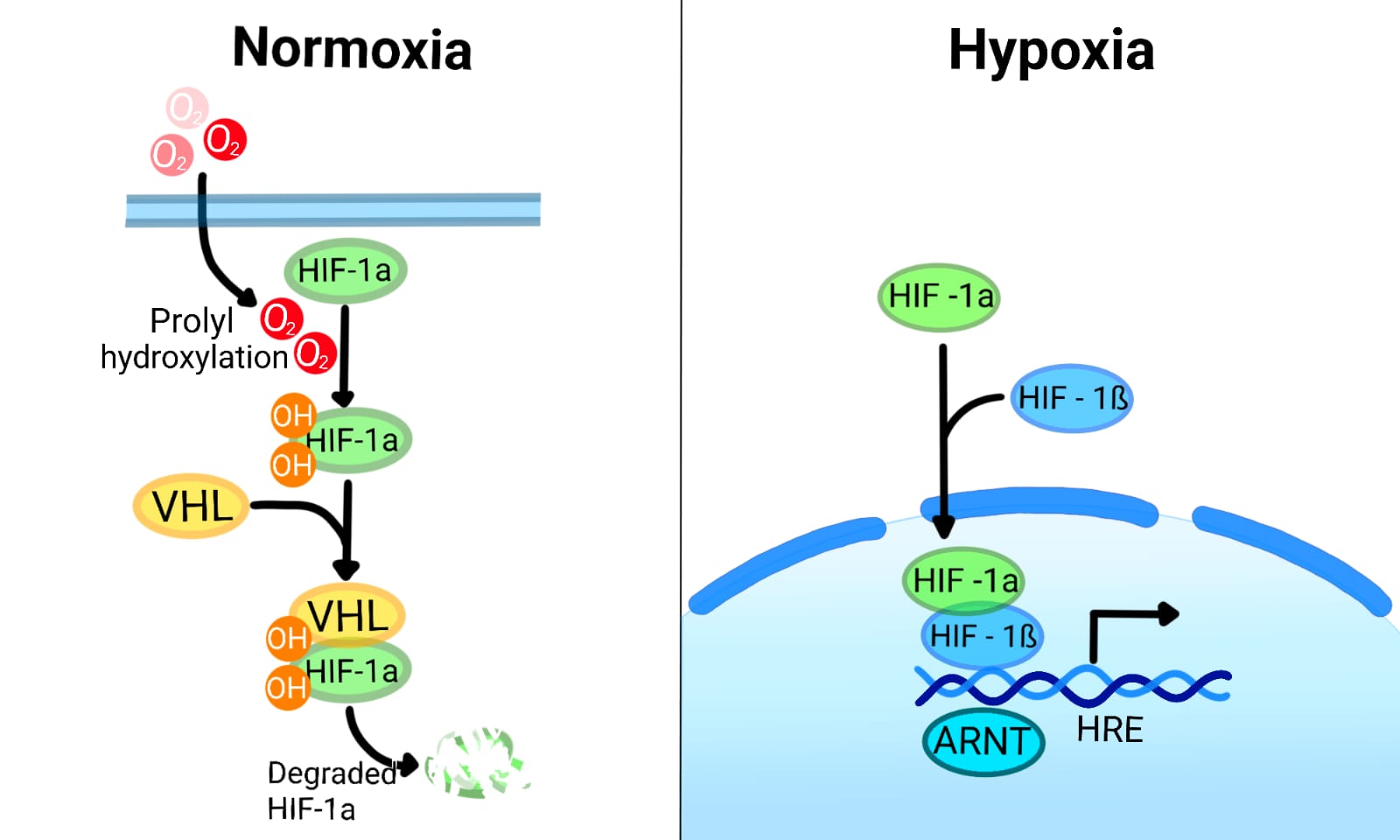


Figure S1: HIF in normoxic and hypoxic conditions. Designed by Kayleigh Evans. Created by Lena Geyer with program Sketchbook Version 6.1.1 by Google Commerce Ltd available over Google Play Store.


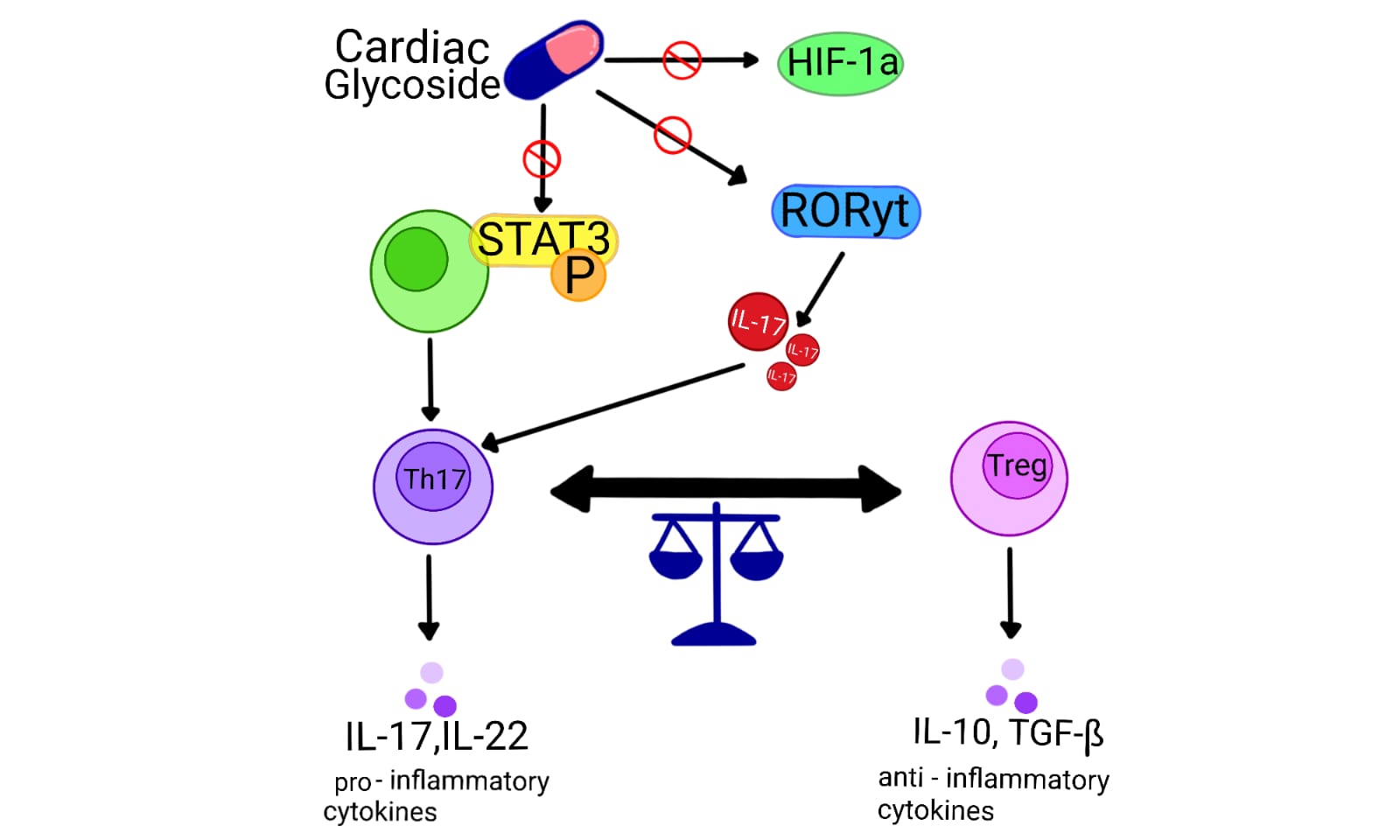


Figure S2: The multiple paths of influence of NKA inhibitors on TH17 cell differentiation. Designed by Kayleigh Evans. Created by Lena Geyer with program Sketchbook Version 6.1.1 by Google Commerce Ltd available over Google Play Store.
